# A Comparison of Walking Behavior during the Instrumented TUG and Habitual Gait

**DOI:** 10.3390/s23167261

**Published:** 2023-08-18

**Authors:** Catherine P. Agathos, Anca Velisar, Natela M. Shanidze

**Affiliations:** The Smith-Kettlewell Eye Research Institute, San Francisco, CA 94115, USA

**Keywords:** timed up and go, walking, gait, instrumented TUG, IMU, motion tracking, head stabilization, postural control, aging, visual impairment

## Abstract

The timed up and go test (TUG) is a common clinical functional balance test often used to complement findings on sensorimotor changes due to aging or sensory/motor dysfunction. The instrumented TUG can be used to obtain objective postural and gait measures that are more sensitive to mobility changes. We investigated whether gait and body coordination during TUG is representative of walking. We examined the walking phase of the TUG and compared gait metrics (stride duration and length, walking speed, and step frequency) and head/trunk accelerations to normal walking. The latter is a key aspect of postural control and can also reveal changes in sensory and motor function. Forty participants were recruited into three groups: young adults, older adults, and older adults with visual impairment. All performed the TUG and a short walking task wearing ultra-lightweight wireless IMUs on the head, chest, and right ankle. Gait and head/trunk acceleration metrics were comparable across tasks. Further, stride length and walking speed were correlated with the participants’ age. Those with visual impairment walked significantly slower than sighted older adults. We suggest that the TUG can be a valuable tool for examining gait and stability during walking without the added time or space constraints.

## 1. Introduction

The timed up and go (TUG) test is a simple, commonly used clinical measure of functional balance, requiring an individual to stand from a seated position, walk 3 m, turn, and sit back down and is scored based on the task duration using a stopwatch [1]. The test is used as a clinical tool for mobility and fall risk screening—though its diagnostic accuracy has been debated [2]. The test has been used diagnostically in a whole host of clinical applications, such as Parkinson’s disease [3,4], physical and mental health factors [5], vestibular dysfunction [6], aging [7], and vision deficits, such as central visual field loss [8,9]. While the test has not been widely adopted in this latter population, it has been suggested as a potentially effective way to assess fall risk in central field loss [10].

While the clinical TUG (where the measured outcome is task duration) may not always be a sensitive enough measure for diagnostic purposes, the task itself is meaningful to daily life and fall risk as it includes several ecological postural transitions (sit-to-stand, gait initiation, turning), i.e., movements more susceptible to loss of balance [11,12], and which therefore require better control. With the rise of lightweight wearable technology, the instrumented TUG is increasingly used to obtain objective, quantifiable postural and gait measures that are more sensitive to mobility changes, for example, in identifying fall risk among older adults or severity in Parkinson’s disease [13,14,15,16]. Despite its own criticisms [17], the instrumented TUG has the potential to become a simple and reliable measure of changes in balance and mobility [18].

Head stabilization is a motor skill, important for providing a stable reference platform for the visual and vestibular systems and to maintain stable gaze [19,20]. To maintain the head stabilized, the trunk and lower limbs act as shock absorbers by attenuating accelerations that would otherwise be experienced by the head [21,22]. Moreover, stabilizing the head in space is primarily driven by vestibular signals [23], and individuals with vestibular dysfunction tend to adopt more rigid head stabilization strategies, blocking the head on the trunk [24], thus experiencing greater head motion since attenuation between the trunk and the head is reduced. With vestibular aging, this rigid stabilization is also more evident during turns [25] and among fall-prone older adults [26]. Visual input also helps to stabilize the head [27,28,29], and there is evidence of reduced head stabilization in visually impaired individuals [30].

Examining head stabilization during the TUG may therefore provide a dual advantage in using this test in older individuals, or those with visual and/or vestibular deficits: on the one hand providing a measure of functional balance and on the other indicating compensatory or adaptive changes in the integration and use of sensory information. It is unknown, however, whether body coordination during the TUG is representative of walking in daily life. We therefore sought to first determine if commonly studied gait metrics (e.g., walking speed, stride length, and duration) during the straight walking portion of the TUG can be used as an analog for normal walking and, second, whether head and trunk accelerations (commonly used to assess stabilization) as measured during the TUG are consistent with those during normal walking. We therefore recorded participants performing the TUG and a simple walking task to compare body segment and gait metrics between these tasks.

One of the barriers to instrumentation during walking tasks is the cumbersome nature of the procedure, with (often frail) participants having to endure a lengthy process of sensor or marker placement on multiple body locations. Prior studies that examined the utility of the instrumented TUG have looked at using as few as a single sensor [31], while others examined the use of a significantly larger number to parse the task as much as possible (e.g., 17 in [32]). In this study, we wanted to understand the utility of instrumenting the participant with a minimal number of motion sensors sufficient to not only record the classic gait metrics but also examine head and trunk stabilization as a metric of functional balance (one on the head, one on the trunk, and one on the ankle of a participant’s leg).

The TUG was initially developed and validated as a clinical assessment of functional balance in older adults [1,33], with more recent studies looking at its usefulness in examining balance and mobility in younger individuals (e.g., [5]). Thus, we recruited participants in two different age categories (younger adults and older adults) to see if the relationship between inertial measurement unit (IMU)-based metrics in TUG and walk differed in these groups. We also recruited a cohort of individuals with visual impairment to determine the discriminative nature of the different metrics available from instrumenting the TUG, as compared to the performance time commonly used.

## 2. Materials and Methods

### 2.1. Participants

All research was performed in accordance with the Declaration of Helsinki and was approved by the Institutional Review Board at the Smith-Kettlewell Eye Research Institute. Further, 40 participants (21 female) were recruited for this study: 30 individuals with healthy vision and 10 with central visual field defect (CVF), and 19 were classified in the younger adults (YA) group (age: 35.0 ± 10.6 years, 9 female), 11 were classified in the older adults (OA) group (age: 72.9 ± 6.6 years, 8 female), and the 10 individuals with CVF were classified in the visually impaired (VI) group (age: 71.0 ± 8.0, 4 female). All participants in the VI group had standing diagnoses of advanced macular deficit in one or both eyes.

Inclusion criteria were that all participants were able to walk unassisted, had no neurological or motor disorders that could affect their ability to complete the task, and no profound hearing loss. Vestibular function was screened using the Dizziness Handicap Inventory [34]. Visual impairment was assessed binocularly using the ETDRS chart for visual acuity and Pelli–Robson chart for contrast sensitivity. Visual field loss was assessed monocularly using a scanning laser ophthalmoscope. None of the participants had any electronic implanted devices, such as pacemakers.

### 2.2. Equipment

**Figure 1:**
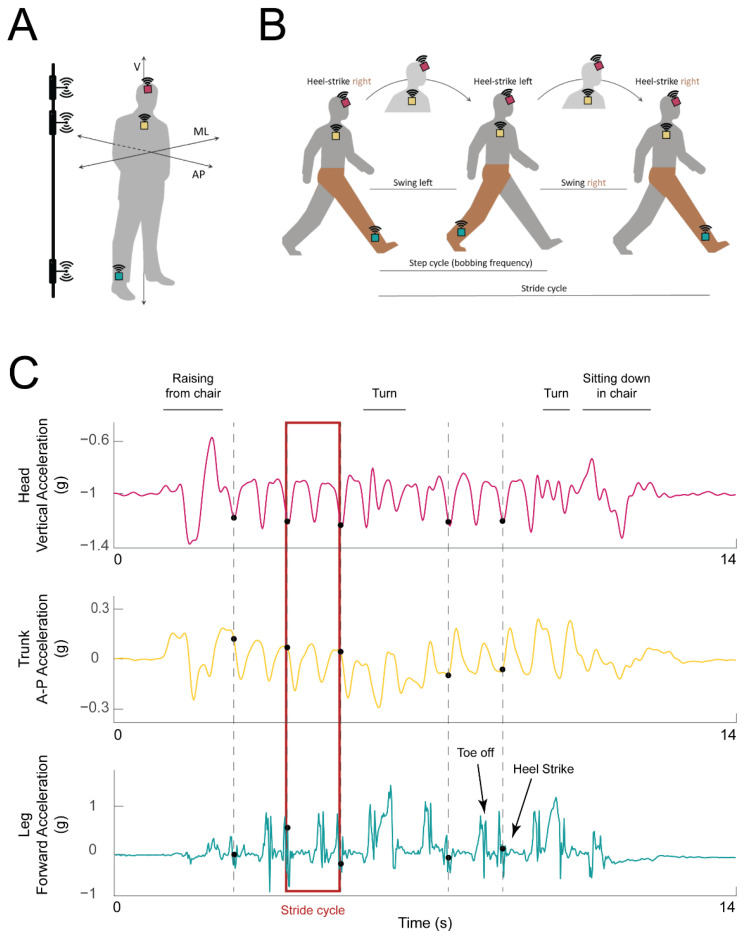
**Experimental setup and example data traces.** (**A**) IMU placement on the ankle (turquoise box), chest (yellow box), and head (magenta box) of the participant. The axes along which acceleration was calculated are marked: vertical (V), mediolateral (ML), anteroposterior (AP). Electromagnetic pulse emitters used for synchronization are represented on the left, attached to a vertical bar. (**B**) Illustration of a single stride cycle of an instrumented participant (IMUs shown as in A). The stride cycle starts with the heel strike of the right (instrumented) foot. During each step, the head travels along the vertical axis (bobs; the highest head position is indicated with the head in lighter gray). The step ends with the left heel strike. At the end of the second step, the right heel again strikes the ground, and the stride cycle is over. The right (instrumented) leg is highlighted in brown for clarity. (**C**) Example acceleration traces from the head (top: vertical acceleration, magenta trace), trunk (middle: forward (AP) acceleration, yellow trace), and leg (bottom: acceleration along the AP axis, turquoise trace). Detected heel strikes are marked with black dots. Example stride cycle is highlighted with a red rectangle.

Participants were equipped with three 9-Axis Inertial Measurement Units (IMUs) with Bluetooth 2.1 and 4.1 connectivity (LPMS-B2, LP Research, Tokyo, Japan) placed a few centimeters above the right ankle, between the sternum and clavicle on the chest, and on the forehead (turquoise, yellow, and magenta squares in Figure 1A, respectively). Each IMU was securely attached using an ankle strap, chest harness, and ratcheting head gear, respectively.

The IMU mounted on the ankle was used for gait event detection—specifically heel strikes—according to prior literature (e.g., [35,36,37,38]). The sensors on the head and chest were placed such that their coordinate frames were aligned with each other and both sensors’ local Z axes were aligned with the heading direction (see more details in Section 2.4 below). Prior studies have used similar IMU placement to study head and trunk movement [39,40]. The signals from the trunk and head were used to understand how the corresponding body segment was moving during that stride (trunk motion was assessed from the trunk IMU and head motion was assessed from the head IMU). The data from the trunk IMU were further used to obtain walking speed and stride length [41], as described in more detail below.

IMU data were recorded using OpenMAT software, version 1.3.5 (LP Research). For each IMU, the angular velocity, linear acceleration, and magnetic field data were recorded over all three axes using a sampling frequency of 50 Hz. The quaternions were provided at the time of recording by the recording software using a sensor fusion algorithm based on only acceleration and angular velocity data filtered with a Kalman filter. In a few cases when the quaternions were not recorded during the experiment, they were calculated offline. To synchronize the three streams of data from the IMUs, we used three weak field electromagnetic pulse emitters connected in parallel (vertical bar, Figure 1A). The EM pulse, lasting 4.5 ms, was detected and recorded by the magnetometers of the IMUs. The EM pulse was triggered using a digital output channel of a Power1401-3A data acquisition board controlled from Spike2 software (Cambridge Electronic Design, CED, Ltd., Cambridge, UK). The EM pulse timing, the event logging, and the synchronized video recording from a webcam (Logitech, Lausanne, Switzerland) were simultaneously recorded and used offline for data synchronization and data parsing.

For six participants, synchronization was instead performed using a fourth (wired) IMU (LPMS-CURS2, LPMS Research), which was tapped against each of the IMUs on the participant in succession. In these cases, the event logging was completed by tapping on the wired IMU. The taps were detected offline as sharp peaks in the acceleration data and used further for parsing the data.

### 2.3. Walking Tasks

Once equipped with the IMUs, participants were asked to perform two tasks: a 3 m timed up and go test (TUG), where they were asked to stand up from a chair, walk 3 m, turn, come back, and sit down in the chair without using the chair armrests (repeated three times), and a loop-walking task (repeated twice). For the latter, participants were asked to walk between two lines on the floor at 4 m apart, in a continuous way such as to complete a wide loop trajectory (3 turns, after passing the floor mark each time). Four visually impaired participants were tested off-site at the Envision Research Institute (Wichita, KS, USA). For these individuals, the walking task was walking along a straight 7 m path, repeated twice. For both tasks, participants were asked to walk at their habitual pace. At the beginning of each task, participants were asked to either sit (TUG) or stand (walk) quietly without moving, facing the heading direction for a few seconds. This quiet stance period was logged as an event.

### 2.4. Data Processing and Analysis

All data processing was performed in MATLAB (MathWorks, Natick, MA, USA). IMU data from the three motion sensors were synchronized offline between the sensors and the event log. The peak in the magnetic field signal recorded by each magnetometer was aligned with the rising edge of the triggering pulse (recorded in Spike2) of the EM pulse emitters. Subsequent events logged in Spike2 software during recording were used to parse the data. In the cases where tapping of a fourth IMU was used for synchronization and event logging, the related peaks in acceleration signals of all IMUs were used to align the data. The subsequent peaks in acceleration signals of the fourth IMU were used to parse the data. The initial orientation of the IMUs was calculated during the quiet stance period described above, using the quaternions, when available. In other cases, the quaternions were calculated using Madgwick algorithm (script: https://github.com/xioTechnologies/Fusion (accessed on 14 August 2023), algorithm details: https://ethos.bl.uk/OrderDetails.do?uin=uk.bl.ethos.681552 (accessed on 14 August 2023)). Both sensor fusion algorithms used only the accelerometer and gyroscope signals as there were ferrous objects in the experiment room. As a result, the orientation of the IMUs in the horizontal plane is not exact. However, we placed the sensors on the head and trunk with their local Z axis oriented with the heading direction. An additional rotation around the global Z axis (gravity axis) was calculated such that the local Z axes of the head and trunk sensors were aligned with the global Y axis, thus aligning them with the heading direction.

**Figure 2:**
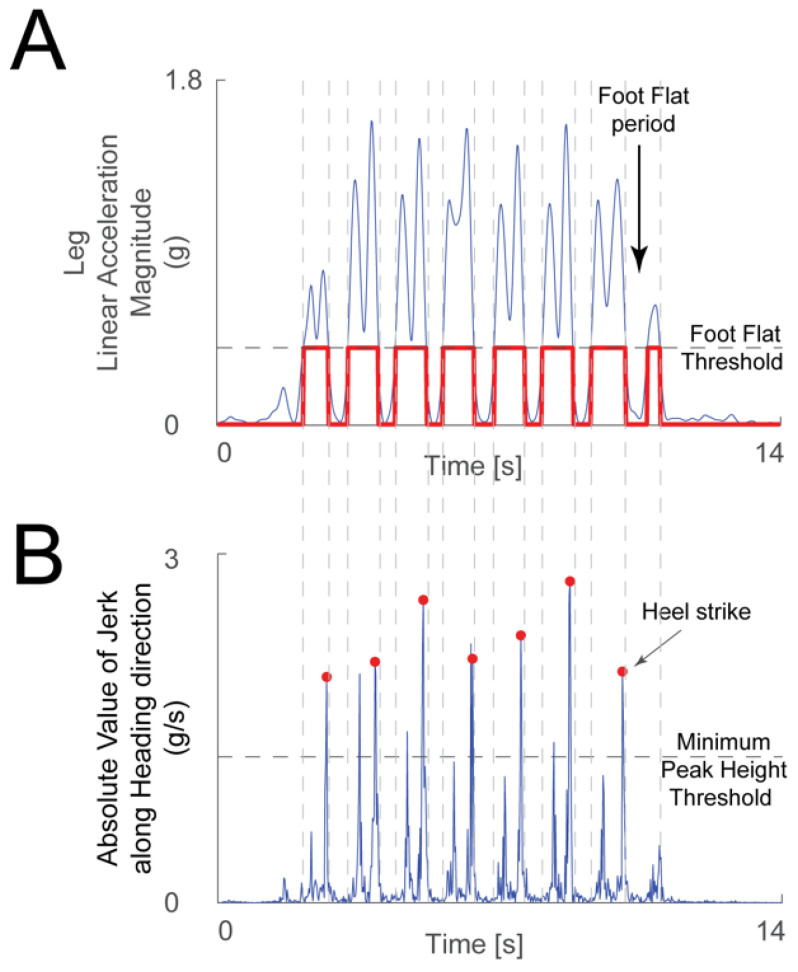
**Heel strike detection method.** (**A**) The linear acceleration magnitude from the leg IMU (blue trace) was used to determine periods when the foot was flat on the floor—when the acceleration was below the foot flat threshold (gray dashed line), and periods where the foot was moving—when the acceleration magnitude was above the threshold (chosen manually for each recording). The logical signal that highlights the foot flat periods is shown in red. (**B**) A peak detection algorithm was used on the absolute value of the jerk signal along the heading direction (blue trace) during the periods when the foot was moving. Toe off and heel strike events create large peaks in the jerk signal during a period of significant foot movement. When the algorithm found more than one peak larger than the minimum peak height threshold (gray dashed line, chosen manually for each recording) in one period, the second peak was chosen as heel strike (red dots).

The linear acceleration signals from the leg IMU were used to detect heel strikes. The acceleration magnitude was low-pass filtered (3 Hz cutoff frequency 2nd order Butterworth filter), and then sections of minimal motion of the foot were detected when the acceleration signal was under a threshold chosen manually for each recording. Heel strikes were detected by differentiating the acceleration signal along the heading direction from the ankle IMU to calculate jerk during periods of leg motion. The heel strikes in this jerk signal were identified as follows. The peaks’ minimum height threshold was chosen manually (Figure 2B). Generally, there are two large peaks in the jerk signal during leg motion: one due to toe off and the second due to heel strike (Figure 2A). The algorithm chose the second peak if both were detected. An experimenter verified by visual inspection that the algorithm picked the correct heel strike events. The time distance between successive heel strikes defined the duration of a stride, or gait cycle.

The IMU data for the whole trial were linearly interpolated for missing samples (<1% of samples on average across all participants) and filtered with a low-pass Butterworth 4th order filter with a 5 Hz cutoff frequency.

The instrumented TUG duration for each participant was calculated as the mean of all TUG trials available for that participant. Each TUG trial duration was calculated from the “start” and “stop” event triggers made at the time of the recording by the experimenter. This method is similar to the stopwatch method used in clinical settings.

The stride cycles that corresponded to straight line walking were chosen manually to exclude gait initiation and termination cycles and turns. Previous studies have shown that steady state gait is achieved within two steps (one cycle) when initiating gait [42,43]; thus, our cycle selection approach allowed us to compare equivalent walking patterns on the TUG and walk tasks.

**Figure 3:**
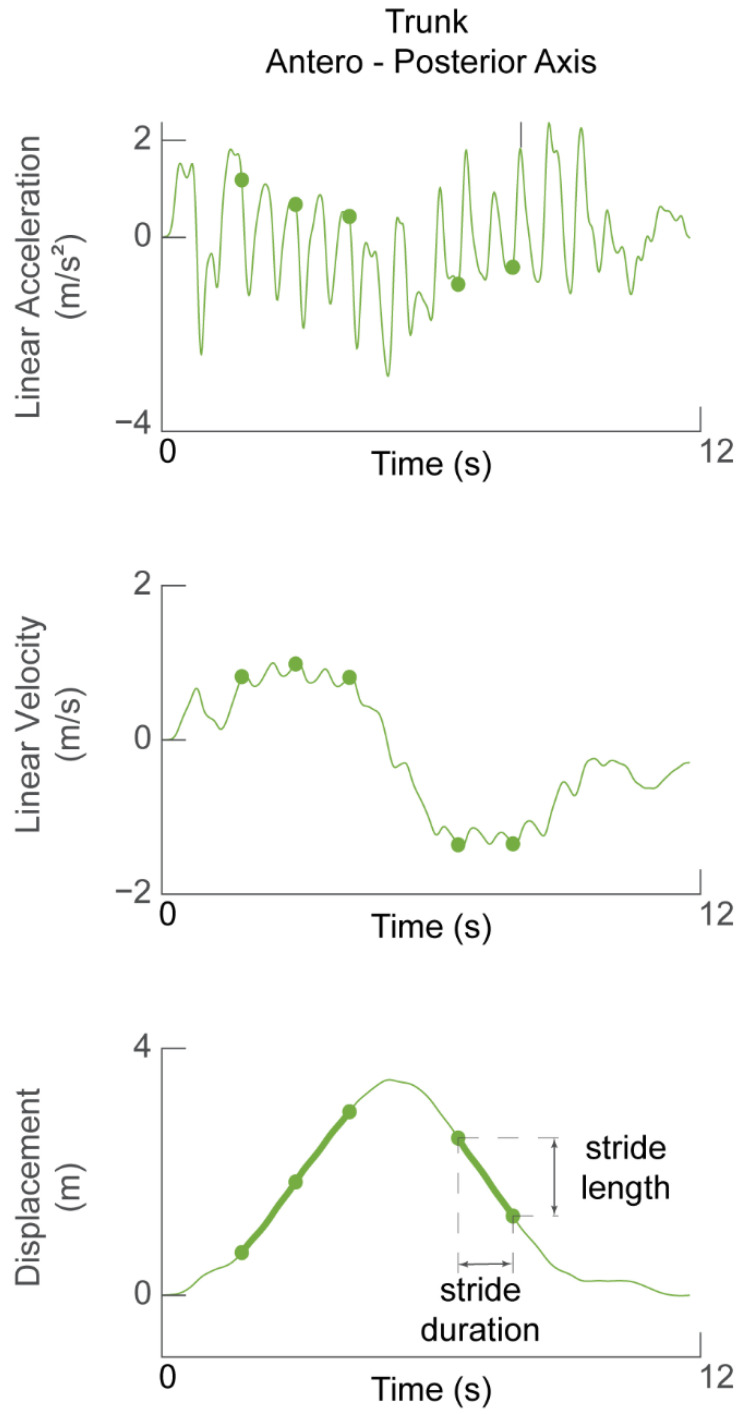
**Determining stride length and walking speed.** Linear acceleration in the forward (AP) direction (**top panel**) during one TUG task was integrated to obtain forward velocity (**middle panel**). The signal was then integrated again to obtain displacement of the participant during one TUG task. Green dots in all panels represent the time of the detected heel strikes and therefore demarcate stride cycles between them. Stride length is defined as the change in trunk displacement during one stride cycle.

The trunk linear acceleration along the heading direction was used to determine stride length and walking speed. The acceleration signal was de-trended and direct-reverse integrated [44] twice within the limits of walk initiation and walk termination in order to obtain the displacement of the trunk along the heading direction (Figure 3). Thus, the accuracy of the displacement estimate was affected by how stationary the participant was at the start and end of the task, i.e., how much the acceleration signal deviated from zero.

The stride length was calculated as the difference in the forward displacement of the trunk at the end of the stride with respect to the beginning (Figure 3, bottom panel). The stride speed was calculated as the ratio of stride length to stride duration. The walking speed was defined as the average of the speed of the stride cycles selected for analysis (i.e., straight walking).

The trunk and head acceleration data were parsed over the chosen cycles. Data of all chosen cycles from a given task were interpolated in time such that all cycles had the same time duration. The average cycle was computed as the per-sample average of the data from all chosen cycles.

Power spectrum for the average cycle was calculated to estimate the peak power and the respective frequency for the head and the trunk segments. The peak frequency of the linear acceleration of the head and trunk along the gravity direction (global Z axis) was defined as the bobbing frequency (Figure 1A). This measure was used as a proxy for step frequency to further analyze walking patterns between the two tasks since vertical acceleration reaches a maximum twice within each stride cycle (during mid-stance on each step). Additionally, the horizontal linear acceleration amplitudes of the average cycle, defined as the ranges along the antero-posterior axis (heading direction) and along the medio-lateral axis (orthogonal to the heading direction in the horizontal plane), were compared between tasks for both head and trunk.

### 2.5. Statistics

Gait (stride duration and length, walking speed, and bobbing frequency) and segmental linear acceleration data were analyzed using MATLAB (2019b version, MathWorks, Natick, MA, USA) and Prism (version 10.0.0, GraphPad, Boston, MA, USA) statistical software. Two-way comparisons were completed using a repeated measures mixed effects model with the Šidák test for multiple comparisons. Group comparisons were completed only along a single dimension—age or visual impairment—meaning that older adults with visual impairment were only compared with the older healthy-sighted group (impairment dimension), and younger healthy-sighted adults were only compared with the older healthy-sighted adults (age dimension). In other words, the older adult group was treated as the control group. A Geisser–Greenhouse correction for non-sphericity was applied where appropriate. Alpha level was set at 0.05.

## 3. Results

### 3.1. TUG versus Walking

To evaluate if a participant walked differently during the straight walking phase of the TUG and a walking task, we first compared the average stride duration of all chosen cycles, the average stride length, and the walking speed between the two tasks for all three groups (younger adults, older adults, and older adults with visual impairment).

#### 3.1.1. Stride Duration

**Figure 4:**
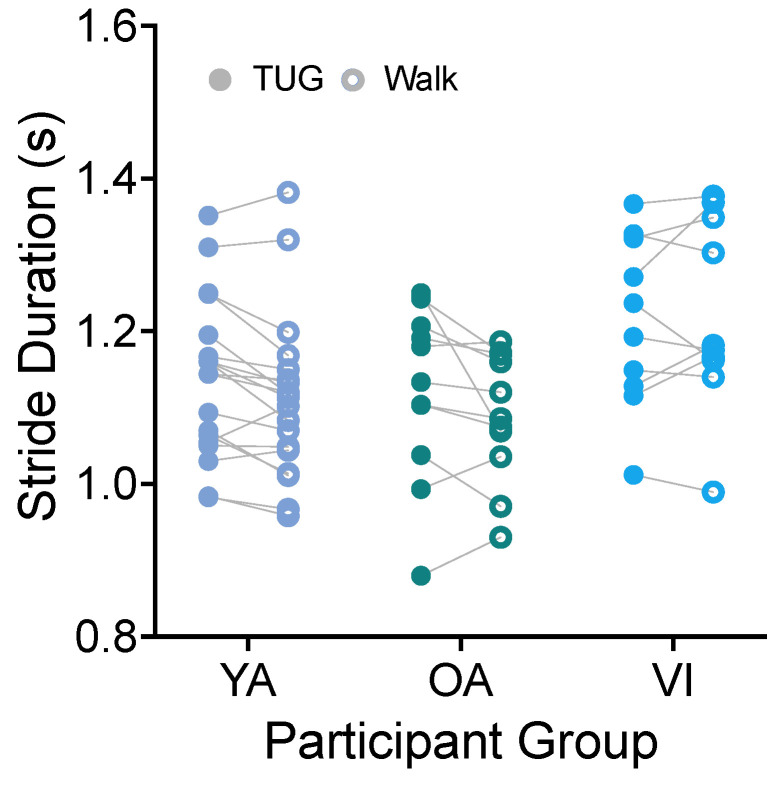
Pairwise comparison of stride durations between the TUG (disk) and walking (circle) tasks for each participant in the younger adult (YA), older adult (OA), and visually impaired (VI) groups. There was no significant difference in stride duration between tasks for all groups.

Using the heel strike detected by the ankle IMU, we directly calculated the stride duration (time from the heel strike of the right leg to the subsequent right leg heel strike) for each participant for each task. Figure 4 shows stride durations compared between tasks for the three groups. There was no significant effect of task for stride duration (Figure 4, F(1, 37) = 4.102, *p* = 0.0501). Because the comparison did approach significance, we provide here the mean difference in stride durations in the two tasks. We found that, on average, stride durations were 185 ms less on the walking task than the TUG.

We did see an effect of participant group (F(2, 37) = 3.511, *p* = 0.0402), with the visually impaired group having significantly longer stride durations than the older adult group (*p* = 0.0372).

#### 3.1.2. Stride Length and Walking Speed

**Figure 5:**
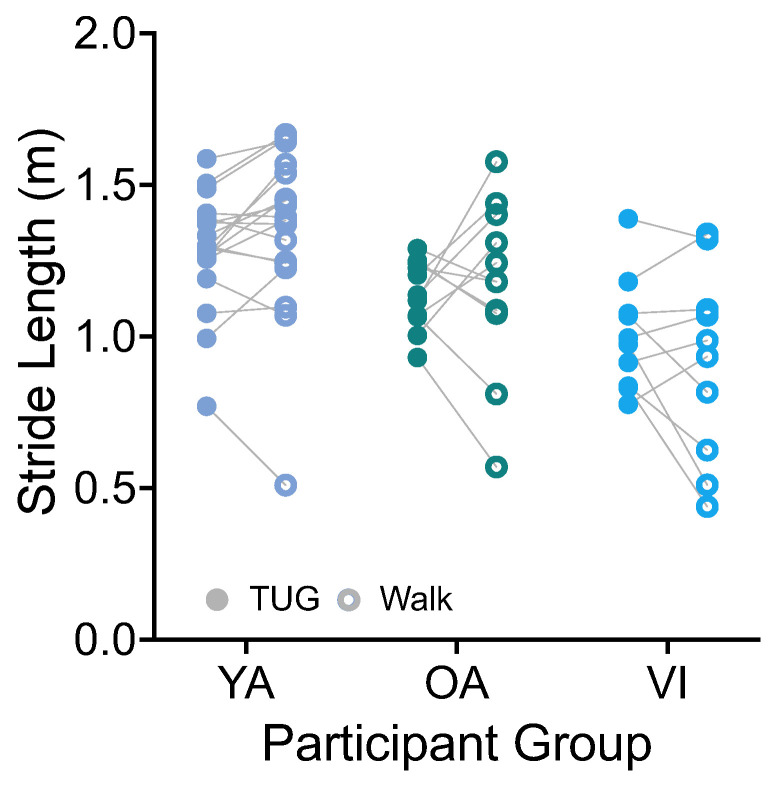
Pairwise comparison of stride lengths between the TUG (disk) and walking (circle) tasks for each participant in the younger adult (YA), older adult (OA), and visually impaired (VI) groups. There was no significant difference in stride length between tasks for all groups.

Stride length was calculated based on the integration of the forward (AP, Figure 1A) trunk acceleration and stride duration. There was no significant effect of task on stride length (Figure 5, F(1, 36) = 0.0001, *p* = 0.9917). There was a significant effect of group (F(2, 36) = 9.141, *p* = 0.0006), which did not persist for the group comparisons of healthy older versus younger, or healthy versus visually impaired older groups (*p* > 0.08).

Given that walking speed is a function of stride length and duration, we predicted that it too would be similar between tasks. Indeed, on average, all groups walked at a similar speed during the TUG and walking tasks (VI: 0.83 ± 0.16 m/s versus 0.77 ± 0.31 m/s; OA: 1.03 ± 0.18 m/s versus 1.08 ± 0.27 m/s; YA: 1.13 ± 0.20 m/s versus 1.21 ± 0.25 m/s; F(1, 36) = 0.532, *p* = 0.4707). The difference between groups was significant (F(2, 36) = 9.679, *p* = 0.0004), with the healthy-sighted older adults walking faster than the visually impaired group (*p* = 0.0190).

#### 3.1.3. Bobbing Frequency (Step Frequency)

**Figure 6:**
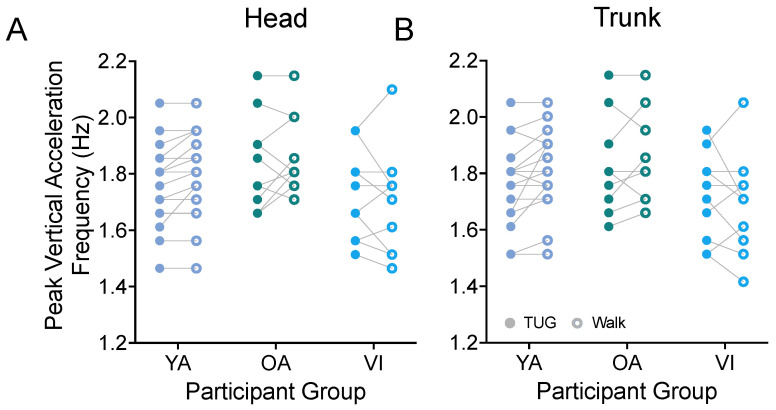
Pairwise comparison of peak frequencies of vertical head (**A**) and trunk (**B**) movements (bobs) between the TUG (disk) and walking (circle) tasks for each participant in the younger adult (YA), older adult (OA), and visually impaired (VI) groups. There was no significant difference in stride bobbing frequencies between tasks for all groups.

Prior metrics focused on the *stride* properties of our participants. While we would have needed to equip both ankles of our participants with IMUs to obtain step properties in the two tasks, we were able to estimate the peak frequency of the vertical acceleration (bob) of the head (Figure 6A) and trunk (Figure 6B) as a proxy for the participants’ cadence and as a way to examine *step* frequency during both tasks. For all groups, we found that the participants had the same stepping frequency between the TUG and walking tasks (head: F(1, 37) = 0.885, *p* = 0.3531; trunk: F(1, 37) = 1.142, *p* = 0.2922).

#### 3.1.4. Head and Trunk Acceleration in the Horizontal Plane

A potential application of instrumenting the TUG could be to look at relative trunk and head motion during the task as a way to measure vestibular function [45] and head/trunk stabilization, for example, as a function of aging [46,47].

**Figure 7:**
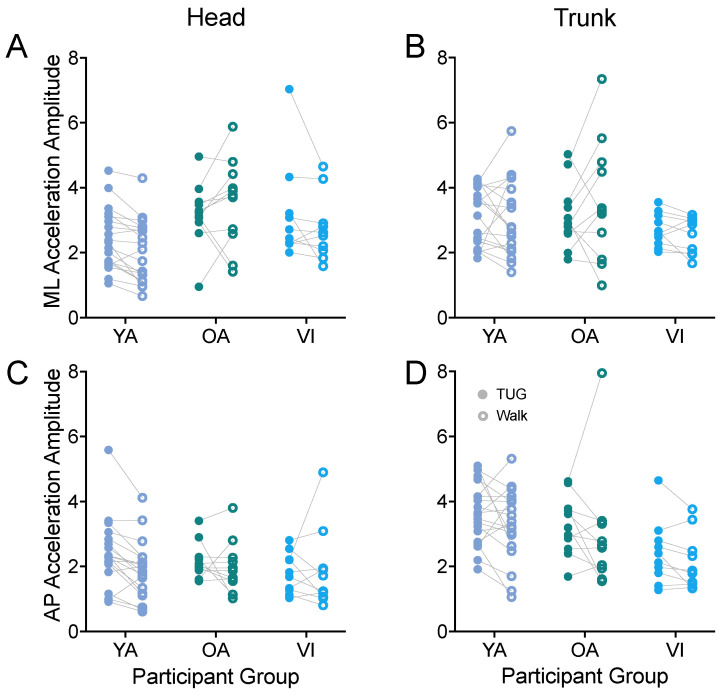
Pairwise comparison of head (**A**,**C**) and trunk (**B**,**D**) accelerations along the ML (**A**,**B**) and AP (**C**,**D**) axes, between the TUG (filled circle) and walking (open circle) tasks for each participant in the younger adult (YA), older adult (OA), and visually impaired (VI) groups. There was no significant difference between tasks for any of the acceleration amplitudes.

Thus, we next examined whether linear acceleration amplitudes along the antero-posterior and medio-lateral axes measured during the TUG agreed with those during the walking task for head (Figure 7A,C) and trunk (Figure 7B,D). First examining the ML axis, we did not observe a difference in head or trunk accelerations between tasks (head: F(1, 37) = 1.456, *p* = 0.2352; trunk: F(1, 37) = 0.111, *p* = 0.7408). We did observe a difference between groups for the head accelerations (F(2, 37) = 4.543, *p* = 0.0172), with the younger adults having significantly lower accelerations than the older healthy-sighted adults (*p* = 0.0125).

Along the AP axis, we also did not observe a difference in head or trunk accelerations between tasks (head: F(1, 37) = 2.492, *p* = 0.1229; trunk: F(1, 37) = 2.622, *p* = 0.1139). We did find a significant effect of group for trunk acceleration (F(2, 37) = 4.636, *p* = 0.0160), which did not persist for the group comparisons of older versus younger, or visually healthy versus visually impaired groups (*p* > 0.09).

We also examined the amplitude of the linear acceleration along the vertical axis. For head accelerations, the amplitudes were similar between the TUG and walking tasks (F(1, 37) = 0.455, *p* = 0.5044). For trunk vertical acceleration amplitudes, there was an overall effect of task (F(1, 37) = 9.669, *p* = 0.0036); the difference did not reach significance for any of the groups when adjusted for multiple comparisons (*p* > 0.1).

### 3.2. Effects of Age on TUG Time and Gait Parameters

**Figure 8:**
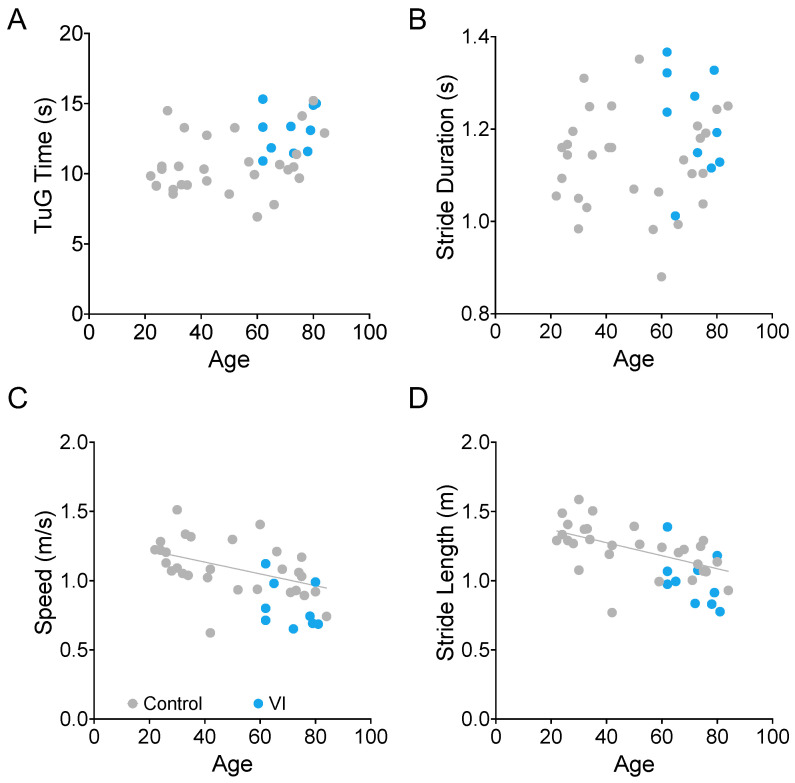
Relationship between age and TUG duration (**A**), stride duration (**B**), movement speed (**C**), and stride length during TUG trials. There is a significant relationship between age and walking speed ((**C**), *p* = 0.0018) and stride length ((**D**), *p* = 0.0001) for the non-visually impaired participants (gray dots). Participants with visual impairment are marked in blue.

We examined the relationship between age and TUG time (Figure 8A), stride duration (Figure 8B), walking speed (Figure 8C), and stride length (Figure 8D). We found that the latter two (walking speed and stride length) were significantly correlated with age in the visually healthy participants (gray dots in Figure 8, Spearman correlation, ρ = −0.555, *p* = 0.0018, ρ = −0.652, and *p* = 0.0001, respectively). The VI group is shown in blue for reference. While this group’s data were consistent with the rest of the sample, they were not included in the age analysis due to the additional confound of visual impairment.

While TUG time itself was not significantly correlated with age, it did have a significant correlation with stride duration (ρ = 0.736, *p* < 0.0001).

## 4. Discussion

The 3 m TUG is composed of several motor components, including sit-to-stand, straight walking, turning, and stand-to-sit portions. In addition to its relevance to motor abilities of daily life, the test is simple to perform, frequently completed in the clinic, and has been used in a number of applications, including research in aging and sensory and motor deficits [3,4,5,6,7,9,48]. While the walking portion itself is quite short, consisting of only a few gait cycles for most individuals, prior studies have shown that individuals achieve their steady-state gait pattern after only two steps (i.e., a single stride, or gait cycle) [42,43], making it potentially feasible to use the TUG to examine gait in this context.

With the increasing availability and decreasing cost of motion tracking options, IMU-based gait analysis has grown significantly [49,50,51,52,53,54] and investigators have leveraged instrumenting the TUG as a way to enhance its predictive value (e.g., [13,14,16,18,55,56], but see also [17]).

In this study, we evaluate the similarity in gait and head/trunk acceleration metrics between the straight walking phase of the timed up and go test and a simple walking task. By doing so, we wanted to establish if IMU-based measurements conducted during the walking portion of the TUG are representative of those same metrics during habitual walking and whether such measurements could be used to assess other behaviors during walking, such as head/trunk acceleration. Because the TUG has different predictive value for groups of different age, fitness, and medical conditions and these groups may find the TUG of variable difficulty, we compared the TUG to walking in three distinct groups—younger adults, healthy-sighted older adults, and older adults with visual impairment.

To that end, we first looked at the similarity of several standard gait metrics to establish if walking was overall similar between the two tasks. Indeed, we find that, both in terms of the timing (stride duration, head and trunk bobbing frequency/step timing) and spatial metrics (stride length, walking speed) we examined, the two tasks were similar for all groups.

Having established that individuals do indeed have similar gait characteristics during the TUG and a simpler walking task, we then examined head and trunk acceleration amplitudes in the horizontal plane, which have been previously used as measures of stabilization and vestibular function (e.g., [46,57]). While individuals had a wide range of head and trunk acceleration profiles, we did indeed find that these tended to be consistent between the straight walking portion of the TUG and the walking task across individuals.

Finally, we wanted to examine the predictive value of the gait metrics on the TUG for age-related changes in walking. We found that, consistent with the walking literature [58], stride length and walking speed were significantly correlated with age, while stride duration was not. Additionally, we found that, in our population, while there may have been a slight trend (ρ = 0.287, *p* = 0.1240), TUG duration and age were not correlated. As mentioned previously, the TUG is made of several different components (sit-to-stand, gait initiation, turning, stand-to-sit, in addition to steady state walking) that may affect overall performance differently. As such, it is the individual’s physical fitness, rather than age, that will manifest in the task duration. Furthermore, we found that TUG duration did significantly correlate with stride duration (which on its own had no relationship with age, consistent with prior work [58]). This result also suggests that time to complete the TUG may be dominated by stride duration to a degree sufficient to reduce TUG’s predictive power.

The current study focuses on the similarities of gait and head/trunk stabilization variables during the steady state gait portion of the TUG and a simple walking task. While our findings are specific to walking in the laboratory, which may differ from everyday overground walking [59], in-laboratory testing of gait is highly valuable in a number of applications (e.g., [60,61,62]). Our results show that the metrics assessed were indeed comparable for the three distinct populations tested. Instrumenting the TUG can, therefore, provide additional metrics of walking behavior beyond those currently used (e.g., [13,16,55,56]), such as head and trunk stabilization. Given our findings, we suggest that future work should examine these variables during other parts of the TUG, including gait initiation and turns. While these are beyond the scope of this study, they may provide additional markers that can be compared to natural behavior and, if similar, be simply and quickly measured in the clinic. Transition tasks, like gait initiation, are health-relevant given their association with increased fall risk [12] and are a more challenging head stabilization condition, even in healthy older adults [63]. Turns, on the other hand, are gaining attention as a more sensitive measure of balance and gait deficits as compared to straight walking (e.g., [64,65]).

## 5. Conclusions

We suggest that the instrumented TUG can be used as a proxy for a walking task to examine head and trunk stabilization, as well as classic gait variables, with the latter being predictive of age and pathology when measured on the TUG [66]. A simple implementation, with three low-cost motion sensors, can thus allow IMU-based measures (shown to have diagnostic value [13,14,16,18,55,56]) to be simply acquired in the clinic or in experimental settings where a longer walk may be too complex or time- and space-prohibitive. Researchers and clinicians can thus expand both the predictive value of the TUG as well as their assessment of gait and body coordination changes that can be observed during normal walking.

## Data Availability

The data presented in this study are available on request from the corresponding author. The data are not publicly available yet due to ongoing studies using datasets, of which this study is a part.
